# Myostatin Inhibition in Muscle, but Not Adipose Tissue, Decreases Fat Mass and Improves Insulin Sensitivity

**DOI:** 10.1371/journal.pone.0004937

**Published:** 2009-03-19

**Authors:** Tingqing Guo, William Jou, Tatyana Chanturiya, Jennifer Portas, Oksana Gavrilova, Alexandra C. McPherron

**Affiliations:** 1 Genetics of Development and Disease Branch, National Institute of Diabetes and Digestive and Kidney Diseases, National Institutes of Health, Bethesda, Maryland, United States of America; 2 Mouse Metabolic Core Laboratory, National Institute of Diabetes and Digestive and Kidney Diseases, National Institutes of Health, Bethesda, Maryland, United States of America; University of Las Palmas de Gran Canaria, Spain

## Abstract

Myostatin (Mstn) is a secreted growth factor expressed in skeletal muscle and adipose tissue that negatively regulates skeletal muscle mass. *Mstn^−/−^* mice have a dramatic increase in muscle mass, reduction in fat mass, and resistance to diet-induced and genetic obesity. To determine how *Mstn* deletion causes reduced adiposity and resistance to obesity, we analyzed substrate utilization and insulin sensitivity in *Mstn^−/−^* mice fed a standard chow. Despite reduced lipid oxidation in skeletal muscle, *Mstn^−/−^* mice had no change in the rate of whole body lipid oxidation. In contrast, *Mstn^−/−^* mice had increased glucose utilization and insulin sensitivity as measured by indirect calorimetry, glucose and insulin tolerance tests, and hyperinsulinemic-euglycemic clamp. To determine whether these metabolic effects were due primarily to the loss of myostatin signaling in muscle or adipose tissue, we compared two transgenic mouse lines carrying a dominant negative activin IIB receptor expressed specifically in adipocytes or skeletal muscle. We found that inhibition of myostatin signaling in adipose tissue had no effect on body composition, weight gain, or glucose and insulin tolerance in mice fed a standard diet or a high-fat diet. In contrast, inhibition of myostatin signaling in skeletal muscle, like *Mstn* deletion, resulted in increased lean mass, decreased fat mass, improved glucose metabolism on standard and high-fat diets, and resistance to diet-induced obesity. Our results demonstrate that *Mstn^−/−^* mice have an increase in insulin sensitivity and glucose uptake, and that the reduction in adipose tissue mass in *Mstn^−/−^* mice is an indirect result of metabolic changes in skeletal muscle. These data suggest that increasing muscle mass by administration of myostatin antagonists may be a promising therapeutic target for treating patients with obesity or diabetes.

## Introduction

Myostatin (Mstn), a member of the transforming growth factor β (TGFβ) superfamily of secreted growth factors, is an important regulator of skeletal muscle development and adult homeostasis. *Mstn* is strongly expressed in skeletal muscle and *Mstn^−/−^* mice have a great increase in muscle mass demonstrating that myostatin is a muscle-specific negative regulator of skeletal muscle size [1], [2]. Mutations in the *Mstn* gene in cattle, sheep, dogs, and one child cause an increase in skeletal muscle mass indicating conservation of function in mammals [3]. Myostatin also regulates muscle mass in adult mice: Inhibition of myostatin by injection of neutralizing antibodies or antagonists causes an increase in skeletal muscle mass in both healthy adult mice and in mouse models of muscular dystrophy [4], [5], [6], [7], [8], [9], [10], [11]. Myostatin inhibitors have therefore generated great interest as candidates for treatment of muscle wasting diseases.

The myostatin protein is synthesized as a full-length precursor that is cleaved into an amino-terminal pro-peptide and a carboxy-terminal mature region which is the active form of the molecule. In skeletal muscle and in circulation, myostatin is found in inactive complexes of differing composition with other proteins such as its own pro-peptide, follistatin-like 3 (Fstl3, also known as follistatin-related gene), and latent TGFβ binding protein [1], [12], [13]. The mechanism of activation of these inactive complexes or whether all of these complexes are capable of being activated is unknown. For complexes containing the pro-peptide, activation likely requires proteolysis of the pro-peptide, perhaps by specific target cells [11], [14]. Once activated, myostatin has high affinity for the activin IIB receptor (Acvr2b, also known as ActRIIB) and weak affinity for Acvr2a (also known as ActRII and ActRIIA), both of which, like other receptors for TGFβ family members, bind multiple ligands [15].

The effects of *Mstn* deletion are not restricted to skeletal muscle. Many skeletal muscles of *Mstn^−/−^* mice are twice the mass of those of *Mstn^+/+^* mice [16] while, in contrast, adipose tissue is greatly reduced in size [17], [18]. Deletion of *Mstn* in genetic mouse models of obesity and diabetes improves obesity and glucose metabolism [18], and *Mstn^−/−^* mice in a CD-1 genetic background are resistant to weight gain due to diet-induced obesity [19]. Furthermore, transgenic mice overexpressing the secreted myostatin pro-peptide antagonist in muscle have increased muscle mass and are resistant to both weight gain and the development of insulin resistance when fed a high-fat diet (HFD) although these mice do not have reduced adiposity or improved insulin sensitivity when fed a standard diet [20].

The *Mstn* gene is expressed at low levels in adipose tissue and myostatin protein is found in circulation suggesting that myostatin could have a direct role in regulating adipocyte differentiation or function [1]. In vitro, myostatin promotes adipogenesis in the multipotential C3H 10T1/2 mesenchymal cell line [21], [22] and inhibits adipogenesis in 3T3L1 preadipocytes [23], [24] indicating that myostatin actions are different during determination and differentiation steps. In vivo, *Mstn* overexpression in adipose tissue results in small immature adipocytes, increased energy expenditure, and resistance to diet-induced obesity [22]. Furthermore, the expression of *Mstn*, *Fstl3*, and *Acvr2b* is upregulated in adipocytes from obese mice suggesting myostatin signaling may play a role in the response of adipocytes to obesity [25].

Whether myostatin directly regulates the overall mass of adipose tissue as it does skeletal muscle, however, is unclear. Experiments using direct injection of myostatin protein have produced conflicting results in regard to the effect on fat mass [24], [26]. Transgenic mice overexpressing *Mstn* specifically in adipose tissue have normal body composition despite a reduction in adipocyte size [22]. In contrast, high systemic levels of myostatin or deletion of the antagonist *Fstl3* cause a loss of adipose tissue mass [24], [27].

Several other transgenic mouse models that have increased muscle mass, including some using muscle-specific promoters to drive expression of genes that cause muscle hypertrophy, also have decreased adiposity demonstrating that changes in adipose tissue size can be indirectly caused by changes in skeletal muscle size [28], [29], [30], [31]. For example, HFD-fed mice expressing a muscle-specific inducible constitutively active *Akt1* transgene lose body and adipose tissue weight after muscle hypertrophy is induced [29]. Thus, a role for myostatin in regulating adipose tissue mass directly cannot be determined by analysis of the extremely muscular *Mstn^−/−^* mouse.

Here, we asked what metabolic changes cause the reduced adiposity and resistance to obesity in *Mstn^−/−^* mice. We then determined whether these changes were mediated by the loss of direct myostatin signaling in adipose tissue, or whether the altered metabolism is due to the loss of myostatin signaling in skeletal muscle.

## Results

### Altered substrate utilization in Mstn^−/−^ mice

One possible explanation for a decrease in adiposity is an increase in metabolic rate. It has been previously shown that *Mstn^−/−^* mice have a slightly higher metabolic rate per animal and slightly lower metabolic rate normalized to total body weight [18]. We reexamined oxygen consumption in *Mstn^−/−^* mice fed a standard diet and normalized the data to lean mass. Our results confirmed that *Mstn^−/−^* mice have a lower metabolic rate when normalized to lean mass or to total body weight (Table S1). Additionally, *Mstn^−/−^* mice had no change in total or ambulatory activity (Table S1). Thus, the improved glucose metabolism in *Mstn^−/−^* mice does not appear to be caused by an increase in metabolic rate or activity.

**Figure 1:**
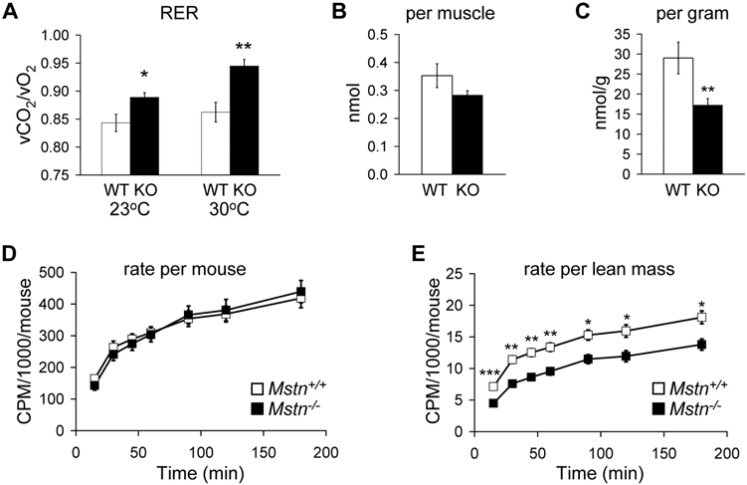
Substrate utilization in *Mstn^+/+^* and *Mstn^−/−^* mice on standard chow. (A) Total respiratory exchange ratio determined by indirect calorimetry. (B) Amount of palmitate oxidized per soleus muscle. (C) Amount of palmitate oxidized by soleus muscle normalized to soleus mass. (D) Rate of oleic acid oxidation per mouse. (E) Rate of oleic acid oxidation normalized to lean mass. *n* = 6–10. *P<0.05, **P<0.01, ***P<0.001.

We also determined the respiratory exchange ratio (RER) in fed mice by indirect calorimetry. The RER is a measure of the ratio of the volume of CO_2_ produced to the volume of O_2_ consumed by an animal which reflects the relative oxidation of carbohydrates to lipids. The RER in fed *Mstn^−/−^* mice was significantly higher than in *Mstn^+/+^* mice suggesting the mutant mice have increased carbohydrate utilization, reduced lipid utilization, or both (Fig. 1A).

To determine whether *Mstn^−/−^* mice fed standard chow had a change in the utilization of lipid, we measured fatty acid oxidation rates in whole animals and in soleus muscle. The rate of fatty acid oxidation per animal was not significantly different between genotypes (Fig. 1D). *Mstn^−/−^* mice had significantly lower rates of fatty acid oxidation compared to *Mstn^+/+^* mice, however, after normalization to lean mass (Fig. 1E). This difference was also seen in isolated soleus muscle. The rate of fatty acid oxidation was not different per muscle (Fig. 1B), but was significantly lower in *Mstn^−/−^* soleus muscle normalized to muscle mass (Fig. 1C). These results suggest that the increase in RER in *Mstn^−/−^* mice is due to increased carbohydrate utilization for energy while overall lipid utilization per animal is unchanged.

### Glucose metabolism in Mstn^−/−^ mice

**Figure 2:**
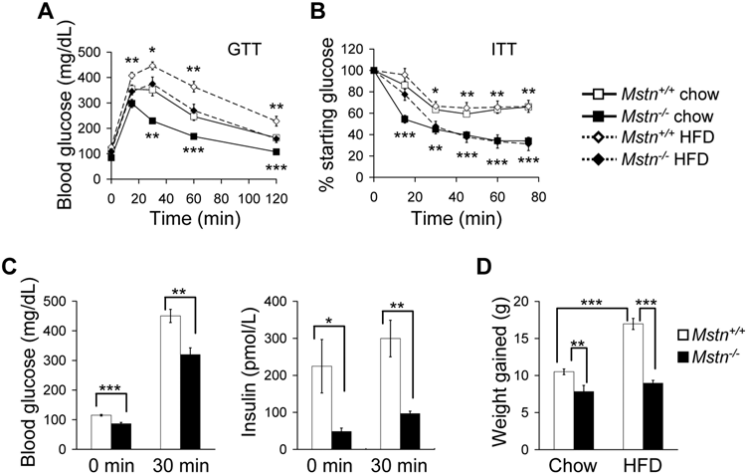
*Mstn^−/−^* mice have increased insulin sensitivity and reduced weight gain on standard chow and HFD. (A) Blood glucose levels during GTT of *Mstn^+/+^* and *Mstn^−/−^* mice on standard chow or HFD. *n* = 8–16. (B) Percent of starting glucose during ITT of *Mstn^+/+^* and *Mstn^−/−^* mice on standard chow or HFD. *n* = 6–10. (C) Blood glucose (left panel) and insulin (right panel) 0 and 30 minutes after glucose injection in fasted mice maintained on standard chow. *n* = 6. (D) Body weight gained by *Mstn^+/+^* and *Mstn^−/−^* mice after 10 weeks on diets. *n* = 7–16. *P<0.05, **P<0.01, ***P<0.001. A and B, *P* value symbols are for comparisons between genotypes on the same diet; below curves, mice on standard chow; above curves, mice on HFD.

The decreased adiposity and increased carbohydrate utilization in *Mstn^−/−^* mice suggest they may have altered glucose metabolism. As previously reported [18], fed and fasting glucose and fed insulin levels in *Mstn^−/−^* mice were not significantly different from controls although insulin tended to be lower (*P* = 0.067) (Table 1). To examine the response to exogenous glucose, we performed glucose tolerance tests (GTT) on mice fed standard chow. *Mstn^−/−^* mice had significantly improved glucose tolerance compared to *Mstn^+/+^* littermates (Fig. 2A). To determine whether the reduced glucose levels in response to exogenous glucose load were due to higher insulin secretion, we measured serum insulin at 0 and 30 minutes after glucose injection in fasted mice. Fasting blood glucose and serum insulin levels were lower in *Mstn^−/−^* mice compared to *Mstn^+/+^* mice 30 minutes after glucose injection suggesting that the improvement in the GTT was due to increased response to insulin rather than to increased insulin secretion (Fig. 2C). We therefore performed insulin tolerance tests (ITT) to measure blood glucose changes after insulin administration. *Mstn^−/−^* mice had lower blood glucose levels than *Mstn^+/+^* littermates after insulin injection demonstrating improved insulin tolerance (Fig. 2B).

**Table 1:** Serum chemistry from mice on standard chow and HFD.

	Fed glucose (mg/dl)	Fasting glucose (mg/dl)	Insulin (pmol/L)	Resistin (ng/ml)	Leptin (ng/mL)	Free fatty acids (mM)	Triglyceride (mg/dl)
**Standard Chow**							
*Mstn^+/+^*	142±5	102±7	544±98	3.78±0.14	24.60±1.32	0.323±0.040	65.3±5.2
*Mstn^−/−^*	140±9	87±5	103±34	3.48±0.13	1.96±0.57‡‡	0.219±0.022	41.1±3.7**
**HFD**							
*Mstn^+/+^*	143±5	126±6†	884±96†	4.79±0.29*	28.34±1.72	0.300±0.020	51.8±5.6
*Mstn^−/−^*	140±7	109±8*	177±50‡‡	3.72±0.26	17.28±3.40†	0.252±0.034	40.3±3.3
**Standard Chow**							
*Non-tg*	140±5	95±3	332±110	3.36±0.08	10.47±2.20	0.479±0.069	61.7±5.4
*Muscle-DN*	124±4	81±4	60±9	3.10±0.20	0.34±0.20	0.222±0.026††	35.7±2.5**
**HFD**							
*Non-tg*	136±4	113±10*	676±107*	4.84±0.50†	18.12±3.56	0.431±0.052	44.5±4.5*
*Muscle-DN*	124±5	89±6	182±50††	3.59±0.14	2.35±0.99**	0.271±0.037	33.6±3.9
**Standard Chow**							
*Non-tg.*	142±5	83±3	346±91	3.79±0.18	10.06±5.81	0.378±0.059	52.5±8.7
*Fat-DN*	146±7	104±10	136±21	4.82±0.12	16.37±3.92	0.181±0.017††	44.4±5.1
**HFD**							
*Non-tg*	180±12‡	144±6‡	652±120*	5.94±0.31‡	24.42±4.23†	0.286±0.034	46.6±9.8
*Fat-DN*	167±5*	115±7	513±139†	8.10±0.76‡,‡‡	23.39±4.48	0.199±0.019	40.5±3.3

Data are expressed as mean±SEM of 5–17 per group.

* *P*<0.05.

† *P*<0.01.

‡ *P*<0.001 vs. same genotype on standard chow.

** *P*<0.05.

†† *P*<0.01.

‡‡ *P*<0.001 vs. control on the same diet.

Non-tg, non-transgenic controls.

### Response to high-fat diet

We determined whether this improvement in glucose metabolism was maintained in *Mstn^−/−^* mice fed a HFD. Mice were fed a HFD for 10 weeks and compared to age-matched littermates fed standard chow. Food intake as a function of body weight was not different between genotypes on HFD (data not shown). As expected, *Mstn^−/−^* mice gained significantly less weight than *Mstn^+/+^* controls on a HFD over the 10 week interval (Fig. 2D). Unlike *Mstn^+/+^* mice, *Mstn^−/−^* mice gained the same amount of body weight on HFD as they did on standard chow over the same time period. *Mstn^−/−^* mice, however, were not completely resistant to the effects of diet-induced obesity. *Mstn^−/−^* mice fed a HFD had increased fat pad mass (Fig. S1) and larger adipocytes (Fig. S2) compared to *Mstn^−/−^* mice fed a standard diet. The weights of individual muscles, in contrast, were unaffected by diet (data not shown).

A HFD caused glucose intolerance in both genotypes relative to their response on standard chow (Fig. 2A). Similar to the results from mice fed a standard chow, however, *Mstn^−/−^* mice on HFD had better glucose tolerance compared to *Mstn^+/+^* mice on HFD (Fig. 2A). *Mstn^−/−^* mice also had better insulin tolerance on HFD compared to *Mstn^+/+^* mice (Fig. 2B). Fed and fasting glucose in *Mstn^−/−^* mice were not significantly different from controls fed a HFD (Table 1). Serum insulin was significantly lower in *Mstn^−/−^* mice fed a HFD demonstrating that less insulin was required to maintain normal glycemia compared to *Mstn^+/+^* mice (Table 1).

To determine whether other circulating molecules relevant to metabolism were affected by *Mstn* deletion in mice on either diet, we measured levels of free fatty acids (FFA), triglycerides, and the adipokines leptin and resistin. Serum triglycerides were significantly lower in *Mstn^−/−^* mice than in *Mstn^+/+^* mice on standard chow while FFA levels were normal (Table 1). As previously reported [18], serum leptin was significantly lower in *Mstn^−/−^* mice on standard chow than in controls. Serum resistin levels were normal. On HFD, the levels of FFA, triglycerides, leptin, and resistin were not significantly different between *Mstn^+/+^* and *Mstn^−/−^* mice. Liver triglyceride concentrations were also significantly lower in *Mstn^−/−^* mice on both diets (Table S2).

### Insulin sensitivity in Mstn^−/−^ mice

**Figure 3:**
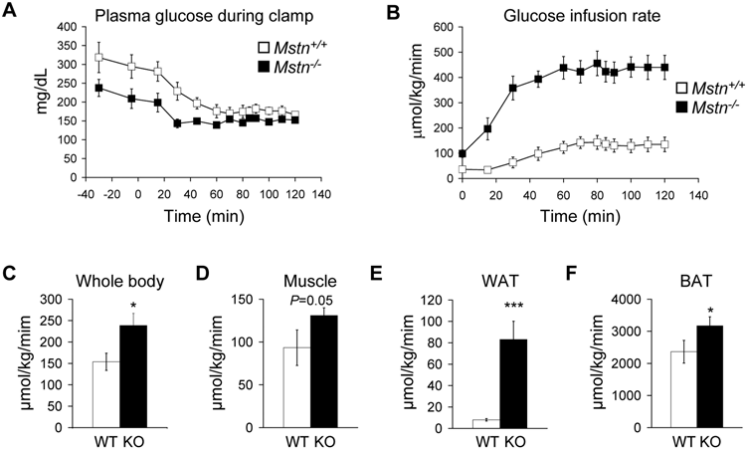
*Mstn^−/−^* mice have increased glucose uptake by hyperinsulinemic-euglycemic clamp. (A) Plasma glucose concentration before and during clamp. Insulin was infused at time 0. (B) Glucose infusion rate during clamp. Glucose uptake in (C) whole body, (D) skeletal muscle, (E) WAT, and (F) BAT. *n* = 7–8. *P<0.05, ***P<0.001.

The improvement in glucose and insulin tolerance, reduction in serum insulin levels, and decrease in adipose mass in *Mstn^−/−^* mice suggest that these mice have increased insulin sensitivity relative to *Mstn^+/+^* mice even on standard chow. To determine whether *Mstn^−/−^* mice have higher glucose uptake than *Mstn^+/+^* mice, we carried out a hyperinsulinemic-euglycemic clamp experiment. In the basal state, *Mstn^−/−^* mice had significantly reduced plasma glucose (Fig. 3A) and insulin levels (189±52 pmol/L in *Mstn^−/−^* mice vs. 361±69 pmol/L in *Mstn^+/+^* mice, *P* = 0.018). Basal glucose turnover, however, was not significantly different between genotypes (89±21 µmol/kg/minute in *Mstn^−/−^* mice vs. 103±15 µmol/kg/minute in *Mstn^+/+^* mice). During the clamp, although insulin levels were raised to ∼688 pmol/L in both strains, the glucose infusion rate (GIR) required to maintain normal glycemia was 181% higher in *Mstn^−/−^* mice than in *Mstn^+/+^* mice indicating that overall insulin sensitivity was improved in the mutant mice (Fig. 3B). Under the clamp conditions used, endogenous glucose production was completely suppressed in both genotypes (data not shown). Whole body glucose uptake in *Mstn^−/−^* mice was significantly higher (Fig. 3C), and the increase in skeletal muscle glucose uptake was nearly significant (Fig. 3D, *P* = 0.053). Glucose uptake was also increased in adipose tissue of *Mstn^−/−^* mice, particularly white adipose tissue (Fig. 3E and F). This result may be at least partially due to an increase in cell number per gram of fat pad because of smaller adipocyte size (Fig. S2). These results demonstrate that *Mstn^−/−^* mice have greatly improved whole body glucose uptake in response to insulin even on standard chow.

**Figure 4:**
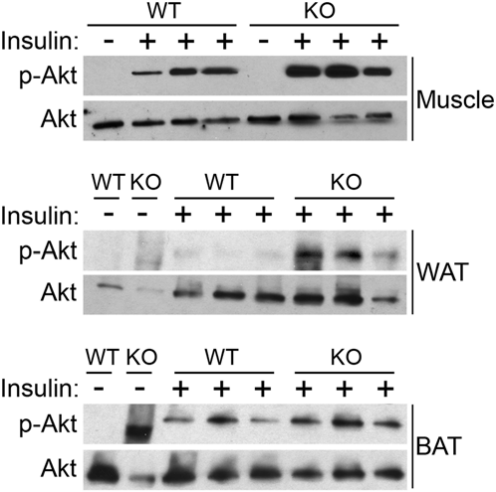
Insulin-stimulated activation of Akt by Western blot. In vivo total Akt and phospho-Akt in gastrocnemius muscle, WAT, and BAT from *Mstn^+/+^* and *Mstn^−/−^* mice with or without insulin stimulation. Each lane contains protein from a different animal.

The increase in glucose uptake in muscle and adipose tissues during the clamp suggests these tissues in *Mstn^−/−^* mice may have enhanced insulin signaling. We injected insulin into fasted mice and assessed activation of the insulin signaling pathway by Western blot (Fig. 4). Insulin signaling normally increases the phosphorylation of Akt as seen in muscle from *Mstn^+/+^* mice. In muscle from *Mstn^−/−^* mice, the level of phosphorylated Akt was higher than in *Mstn^+/+^* mice in response to insulin. Similarly, Akt was also activated to a greater extent in response to insulin in WAT and BAT from *Mstn^−/−^* mice compared to control mice demonstrating enhanced in vivo insulin signaling in mutant skeletal muscle and adipose tissues.

### Tissue-specific inhibition of myostatin signaling

**Figure 5:**
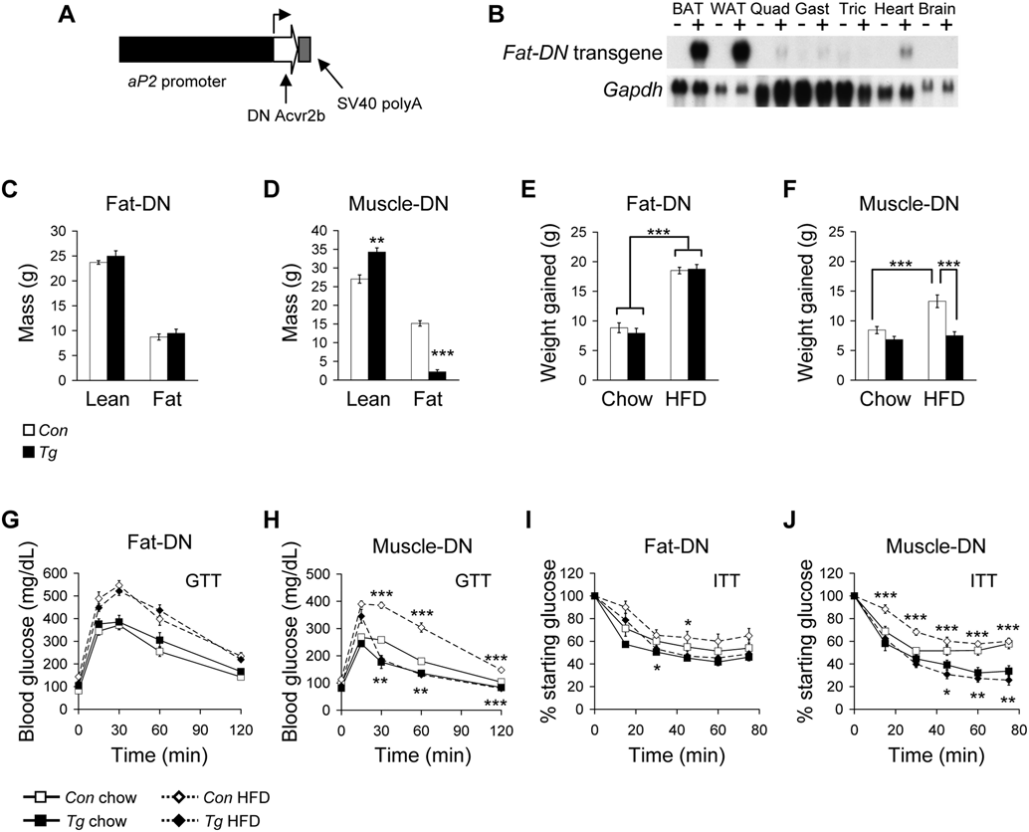
Tissue-specific inhibition of myostatin signaling. (A) Diagram of the construct used to make *fat-DN* transgenic mice with the *aP2* promoter controlling expression of a truncated *Acvr2b* containing the extracellular ligand binding and transmembrane domains. (B) Northern blot analysis of expression of *fat-DN* transgene and *Gapdh* loading control from different tissues from non-transgenic (−) and transgenic (+) mice. Body composition of (C) *fat-DN* (*n* = 10–11) and (D) *muscle-DN* (*n* = 5) male mice compared to non-transgenic littermates. Lean and fat mass are shown as absolute values. Body weight gained by (E) *fat-DN* (*n* = 9–17) and (F) *muscle-DN* (*n* = 9–14) male mice and littermate controls after 10 weeks on diets. Blood glucose levels during GTT of (G) *fat-DN* (*n* = 7–8) and (H) *muscle-DN* (*n* = 9–14) male mice and littermate controls on standard chow and HFD. Percent of starting glucose during ITT of (I) *fat-DN* (*n* = 7–8) and (J) *muscle-DN* (*n* = 9–12) male mice and littermate controls on standard chow and HFD. *P<0.05, **P<0.01, ***P<0.001. G–J, *P* value symbols are for comparisons between genotypes on the same diet; below curves, mice on standard chow; above curves, mice on HFD.

The metabolic changes we found in *Mstn^−/−^* mice could be due to the loss of myostatin signaling in adipose tissue as well as skeletal muscle. To determine whether inhibition of myostatin signaling in adipose tissue alters adipose tissue mass and glucose metabolism independent of increased skeletal muscle mass, we generated a transgenic mouse line expressing a dominant negative (DN) *Acvr2b* gene specifically in adipocytes. An *aP2* (fatty acid binding protein 4) promoter fragment, previously used to control expression of transgenes in differentiated adipocytes [32], was inserted upstream of the *Acvr2b* coding sequence consisting of the extracellular ligand-binding and transmembrane domains without the intracellular kinase domain (Fig. 5A). This construct was used for pronuclear injections. Northern blot analysis of RNA from tissues obtained from mice expressing the adipose-specific dominant negative transgene (*fat-DN*) showed high transgene expression in white and brown adipose tissue (Fig. 5B). A low level of transgene expression was detectable in skeletal muscle either from leaky transgene expression in muscle cells or possibly from intermuscular adipocytes. Heart tissue similarly had low transgene expression. No signal was detectable in brain, liver, kidney, and testes (Fig. 5B and data not shown).

We compared *fat-DN* mice to mice expressing the same dominant negative receptor specifically in skeletal muscle (*muscle-DN*) which have an increase in muscle mass comparable to *Mstn^−/−^* mice [33]. We detected some expression of the transgene in BAT in *muscle-DN* mice (Fig. S3). This expression, however, was considerably lower than the expression of the transgene in *muscle-DN* skeletal muscle or in *fat-DN* BAT (Fig. S3 and 5B). Both the *fat-DN* and *muscle-DN* mice, unlike *Mstn^−/−^* mice, still secrete myostatin into circulation (data not shown). We first measured body composition in each line to determine whether fat mass was affected by these transgenes. We did not detect any differences in body composition in *fat-DN* mice compared to controls (Fig. 5C) although transgenic mice tended to have higher body weight at older ages (data not shown). By histology, the adipocyte cell sizes were similar between *fat-DN* and non-transgenic littermates (Fig. S2). In contrast, *muscle-DN* mice, like *Mstn^−/−^* mice, had an increase in lean mass and a decrease in fat mass compared to non-transgenic littermates (Fig. 5D). Despite having intact myostatin signaling in adipose tissue, *muscle-DN* mice had smaller adipocytes than control littermates, as did *Mstn^−/−^* mice (Fig. S2).

These results suggest that the decrease in fat mass in *Mstn^−/−^* mice is an indirect effect of increased muscle mass or loss of myostatin signaling in muscle rather than a direct effect of the loss of myostatin signaling in adipose tissue. It is possible, however, that a role for myostatin and/or Acvr2b in regulating adipose tissue size might only be seen under conditions that lead to increased adiposity. To test this hypothesis, mice from both transgenic lines were placed on HFD. After 10 weeks, there was no difference in body weight gained between *fat-DN* mice and non-transgenic littermates on standard chow or HFD (Fig. 5E). Liver triglyceride concentrations were also similar between *fat-DN* and control mice on both diets (Table S2). Furthermore, there was little or no difference in glucose or insulin tolerance between *fat-DN* and control mice on either diet (Fig. 5G and I) demonstrating that myostatin inhibition in adipocytes does not affect whole body glucose metabolism under these conditions.

We also measured adipokines and other serum parameters. *Fat-DN* mice had lower circulating free fatty acid levels than control mice on standard chow (Table 1). Serum resistin was higher in *fat-DN* mice which was significant in mice on HFD. These results suggest that myostatin and/or other Acvr2b-binding ligands may have a role in adipocyte function. All other values were not significantly different from control mice.

In contrast to *fat-DN mice*, *muscle-DN* mice were resistant to weight gain due to diet-induced obesity and did not gain more weight on HFD than on standard chow (Fig. 5F) similar to *Mstn^−/−^* mice (Fig. 2D). *Muscle-DN* mice also had improved glucose and insulin tolerance compared to controls on both diets (Fig. 5H and J). *Muscle-DN* mice had lower insulin and leptin levels compared to littermate control mice, which were significant on HFD (Table 1). *Muscle-DN* mice also had lower serum triglyceride and FFA levels on standard chow and tended to have lower liver triglyceride concentrations compared to control mice on both diets (Table S2). Overall, these results demonstrate that *muscle-DN* mice have a similar metabolic profile to *Mstn^−/−^* mice, and that inhibition of myostatin signaling specifically in skeletal muscle, but not adipose tissue, results in reduced adipose tissue mass and ameliorates the consequences of diet-induced obesity.

### Adaptation to increased muscle mass in Mstn^−/−^ mice

**Figure 6:**
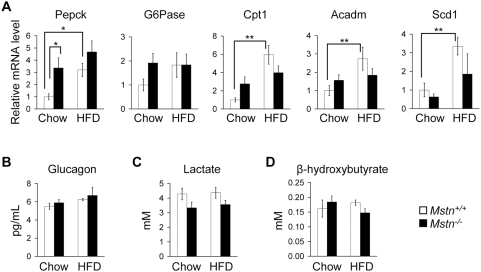
Adaptation to increased muscle mass. (A) Relative mRNA expression levels in liver of *Pepck*, *G6Pase*, *Cpt1*, *Acadm*, and *Scd1* measured by quantitative RT-PCR in *Mstn^+/+^* and *Mstn^−/−^* mice on standard chow or HFD. (B) Serum glucagon, (C) lactate, and (D) β-hydroxybutyrate levels in *Mstn^+/+^* and *Mstn^−/−^* mice on standard chow and HFD. *n* = 6–12. *P<0.05, **P<0.01.

We next asked whether *Mstn^−/−^* mice have similar metabolic responses to increased muscle mass as HFD-fed mice expressing a muscle-specific inducible constitutively active *Akt1* transgene. First, we examined *Mstn^−/−^* mice for evidence of an increase in gluconeogenesis and glycolysis relative to control mice. Constitutively active *Akt1* mice have an increase in expression in liver of genes encoding enzymes involved in gluconeogenesis, phosphoenolpyruvate carboxykinase (*Pepck*) and glucose 6-phosphatase (*G6Pase*), and an increase in serum levels of glucagon, which stimulates gluconeogenesis [29]. We found an increase in expression of *Pepck* in the liver of *Mstn^−/−^* mice relative to control littermates on standard chow but not HFD (Fig. 6A). The expression of *Pepck* in *Mstn^+/+^* mice on HFD was significantly higher than in *Mstn^+/+^* mice on standard chow (Fig. 6A). *Mstn^−/−^* mice, however, did not have increased expression of *Pepck* when fed a HFD relative to standard chow (Fig. 6A). There was no statistically significant difference in the expression of *G6Pase* in liver between genotypes on either diet although the level of *G6Pase* expression appeared to be higher in *Mstn^−/−^* mice than in controls on standard chow (Fig. 6A). Furthermore, there was no difference in serum glucagon levels between *Mstn^+/+^* and *Mstn^−/−^* mice on standard chow or HFD (Fig. 6B) or in basal endogenous glucose production on standard chow as measured prior to insulin infusion during the hyperinsulinemic-euglycemic clamp experiment (89±21 µmol/kg/minute in *Mstn^−/−^* mice vs. 103±15 µmol/kg/minute in *Mstn^+/+^* mice). Constitutively active *Akt1* transgenic mice fed a HFD also have an increase in serum lactate levels, the end product of anaerobic glycolysis, but *Mstn^−/−^* mice did not have increased serum lactate levels compared to *Mstn^+/+^* mice on either diet (Fig. 6C).

We next looked for evidence of altered metabolism of fatty acids in the liver. *Mstn^−/−^* mice have a lower concentration of liver triglycerides than *Mstn^+/+^* mice on both diets (Table S2). The expression of liver carnitine palmitoyl transferase-1 (*Cpt1*), an enzyme required for fatty acid import into mitochondria, and medium chain acyl-Coenzyme A dehydrogenase (*Acadm*, also known as *Mcad* and *Acad1*), an enzyme that metabolizes medium chain fatty acids, are increased in liver from HFD-fed constitutively active *Akt1* mice [29]. We found a non-significant increase in expression of *Cpt1* in *Mstn^−/−^* mice compared to *Mstn^+/+^* mice on standard chow (*P* = 0.085), but there were no differences in expression of *Acadm* between genotypes on either diet (*P* = 0.321 on standard chow and *P* = 0.161 on HFD) (Fig. 6A). Constitutively active *Akt1* transgenic mice on HFD also have higher serum ketone levels indicating increased fatty acid oxidation by the liver [29]. We therefore measured the concentration of the ketone β-hydroxybutyrate in serum from *Mstn^+/+^* and *Mstn^−/−^* mice on standard chow and HFD. There was no increase in β-hydroxybutyrate levels in *Mstn^−/−^* mice on either diet compared to control mice (Fig. 6D). Finally, the expression of stearoyl-Coenzyme A desaturase 1 (*Scd1*), which catalyzes the conversion of saturated fatty acids to monounsaturated fatty acids, is decreased in the liver of constitutively active *Akt1* transgenic mice on HFD reflecting a reduction in lipid synthesis. We did not find any difference in expression of *Scd1* between *Mstn^+/+^* and *Mstn^−/−^* mice on either diet (Fig. 6A). These results demonstrate that the response to diet-induced obesity is different between *Mstn^−/−^* mice and constitutively active *Akt1* mice.

## Discussion

Our results show that *Mstn^−/−^* mice have improved glucose metabolism and increased insulin sensitivity on both standard chow and high fat diet. We also show that the changes in glucose metabolism and adiposity are a consequence of alteration of skeletal muscle rather than a direct effect of the inhibition of myostatin signaling in adipose tissue. Although the adipocyte- and muscle-expressed transgenes showed faint expression in muscle and adipose tissue, respectively, the phenotypes of the mice were very different. Thus, it is unlikely that the leaky expression accounts for the phenotypes presented here.

Like transgenic mice overexpressing myostatin in adipose tissue [22], inhibition of Acvr2b function in adipocytes did not alter body composition. Taken together, the data suggest that the loss of adipose tissue in mice with high serum myostatin caused by a myostatin-secreting tumor [24] required supraphysiologic levels of myostatin. Because the *aP2* promoter we used to express the dominant negative receptor is expressed relatively late in adipogenesis, it remains possible that inhibition of myostatin signaling in very early stage progenitors would have an effect on overall adipose mass. This may be unlikely because, in adipose-specific *Mstn* transgenic mice, no change in body composition was detected even though myostatin secreted by mature adipocytes would in theory be capable of signaling on uncommitted and undifferentiated progenitors within the fat pad [22]. It is also possible that, although Acvr2b inhibition in skeletal muscle phenocopies *Mstn* deletion, multiple ligands with opposite rather than redundant functions bind Acvr2b in mature adipocytes blunting an effect of inhibition of myostatin alone.

The decrease in serum free fatty acid levels in *fat-DN* mice suggests some function for myostatin and/or other Acvr2b-binding ligands in mature adipocytes. However, we and others were not able to see any change in the rate of lipolysis in adipocytes in vitro in response to myostatin [26], and data not shown]. Other metabolic challenges to *fat-DN* mice may reveal specific functions for Acvr2b ligands in adipocytes.

Given our results presented here and the reduction in adiposity in transgenic mice with increased muscularity caused by changes in other signaling pathways such as IGFI [28], [29], [30], [31], we hypothesize that the decrease in adipose tissue size in *Mstn^−/−^* and *muscle-DN* mice is most likely an indirect result of the increased muscle mass. It is also possible the myostatin signaling pathway might have a direct role in regulating muscle metabolism that consequently affects adipose tissue mass separate from its role in regulating muscle mass. In this regard, some in vitro studies have suggested a role for myostatin in regulation of Akt activation. For example, myostatin has been shown to inhibit activation of Akt in phenylephrine-treated cardiomyocytes [34] and reduce the basal level of phosphorylated Akt in C2C12 myotubes [35]. Myostatin also reduces the level of Akt activation in C2C12 undifferentiated myoblasts engineered to overexpress *Akt* [36]. In contrast, myostatin increases basal glucose uptake in human placental extracts [37]. It will be important to examine other signaling pathways in myostatin-deficient skeletal muscle that might regulate metabolism to determine whether the improved metabolism is due to increased muscle mass, the loss of myostatin signaling, or both.

Another plausible explanation for reduced adipose tissue mass in muscular mice is “substrate steal” by skeletal muscle [38]. If skeletal muscle has increased glucose uptake, fewer carbohydrates would be available as substrates for liver triglyceride synthesis and subsequent storage in adipocytes. In this regard, induction of constitutively active *Akt1* caused fast glycolytic fiber hypertrophy in mice on HFD, an increase in glucose uptake into muscle, and an increase in lipid oxidation in liver [29]. *Mstn^−/−^* mice also have fiber hypertrophy in muscles comprised of predominantly fast glycolytic fibers [16], [39]. In addition, *Mstn^−/−^* mice are considerably more muscular than constitutively active *Akt1* mice, have a higher number of fast glycolytic fibers 8, 16, and have less adipose mass even on standard chow [17], [18]. It might therefore be expected that deletion of the *Mstn* gene would result in even more dramatic effects on liver metabolism. We did not find this to be the case although the gene expression data from liver from *Mstn^−/−^* mice fed a standard chow suggest a slight trend toward reduced lipogenesis, increased gluconeogenesis, and increased lipid oxidation (Fig. 6). This trend was not, however, seen in liver from mice fed a HFD unlike in constitutively active *Akt1*-expressing mice fed a HFD [29]. Furthermore, consistent with our data, Stolz et al [26] found no difference in the rate of fatty acid oxidation in the liver of *Mstn^−/−^* mice fed standard chow.

There are several differences between the constitutively active *Akt1* and *Mstn^−/−^* mouse models which might explain why these two muscular mutants did not have the same changes in liver gene expression and in serum values when fed a HFD. By approximately weaning age, *Mstn^−/−^* mice are more muscular than *Mstn^+/+^* mice due to both hypertrophy and hyperplasia of muscle fibers. In contrast, constitutively active *Akt1* transgenic mice gain muscle due to hypertrophy of existing fibers only after transgene induction in adulthood. More subtle compensatory metabolic changes could already have occurred in *Mstn^−/−^* mice in prenatal or early postnatal life. Furthermore, the altered signaling pathways that result in muscle hypertrophy are different in each model. Basal levels of activated Akt are high in constitutively active *Akt1* transgenic muscle [29], but not in *Mstn^−/−^* muscle (Fig. 4). Consequently, signaling to other tissues by myokines or muscle metabolites may be different. Like induction of constitutively active *Akt1*, postnatal inhibition of myostatin signaling in mice causes fiber hypertrophy without hyperplasia or change in fiber composition [8], [9]. Postnatal inhibition of myostatin signaling in mice with diet-induced obesity will be necessary to directly compare these two muscle hypertrophy models to determine whether the reduction in adipose tissue mass in HFD-fed constitutively active *Akt1* mice is caused by hypertrophy of fast glycolytic fibers, by increasing the activation of the Akt signaling pathways, or both.

Injection of a myostatin neutralizing monoclonal antibody in mice on standard chow, however, does not cause a reduction in adipose tissue mass even though the weight of individual muscles is increased 20–30% [9]. Similarly, a milder increase in muscle mass caused by injection of the same monoclonal antibody in the extremely obese leptin receptor-deficient mice does not reduce adipose tissue mass [26]. Mice null for both *Mstn* and *leptin* have a mild decrease in adipose tissue compared to *leptin* null mice, but this improvement is found only in young animals [18]. These results suggest that mice deficient in leptin signaling might be too obese to be rescued by muscle hypertrophy after the onset of obesity. Treatment of mice that are obese due to different genetic or dietary causes with myostatin antagonists will clarify which, if any, specific types of obesity are rescued by postnatal inhibition of myostatin. Additionally, varying the degree of muscle hypertrophy by treatment with different doses of myostatin antagonists will help determine whether there is a threshold level of hypertrophy required for a reduction in obesity.

In addition to the potential for reducing adiposity, we believe that the improved insulin sensitivity in *Mstn^−/−^*, *muscle-DN*, and constitutively active *Akt1* mice suggests that altering metabolism by increasing muscle mass might be a promising strategy for treating diabetes and the metabolic syndrome. Skeletal muscle of diabetics has reduced insulin sensitivity [40], and recent clinical trials suggest that resistance training improves insulin sensitivity in diabetic patients [41], [42]. Similar to the muscle phenotype caused by postnatal myostatin inhibition or inducible constitutively active *Akt* expression [1], [2], [29], resistance exercise causes an increase in fiber size [43]. Some studies have shown, however, that resistance training in humans can also cause shifts in fiber type not seen in these mouse models of postnatal muscle hypertrophy, and mouse muscle contains relatively more fast fibers than human muscle [43]. Nevertheless, it is possible that increasing muscle mass by pharmacologic interventions such as inhibiting myostatin signaling may improve glucose metabolism in diabetics, particularly those in poor physical condition who are unable to perform resistance exercise.

## Materials and Methods

### Ethics Statement

All animals were handled in strict accordance with good animal practice as defined by the relevant national and/or local animal welfare bodies, and all animal work was approved by the Animal Care and Use Committee of the NIDDK, NIH.

### Generation and maintenance of animals

Generation N6 *Mstn^+/+^* and *Mstn^−/−^* mice [16] in the C57BL/6Ncr genetic background were obtained from homozygous matings for all experiments except diet experiments in which N6 *Mstn^+/+^* and *Mstn^−/−^* mice were obtained from heterozygous matings. *Mstn* genotypes were determined by PCR as described [44]. The muscle-specific dominant negative *Acvr2b* transgenic line, C11, in the C57BL6/Ncr genetic background (>N10) has been previously described [33]. *Muscle-DN* mice were genotyped by PCR (forward, 5′-CCATTTATAGTCTGAGCCCGAATGCC-3′; reverse, 5′-AATTGAAGTCATCTAGCCAGCAGCCC-3′; 500 bp). For generation of adipose-specific dominant negative *Acvr2b* transgenic mice, the same truncated mouse *Acvr2b* coding sequence which was used for generation of line C11 (amino acids 1–174) was inserted into the *Pst*I site of KS/422-Flag Ø10 3heptad-F/splice *aP2* promoter vector [32] after removal of the Ø10 3heptad-F insert. Founders in the FVB genetic background were backcrossed to C57BL/6Ncr at least 5 times to generate animals for experiments and were genotyped by PCR (forward, 5′-GCTGCTGCGAAGGCAACTTCTGCAACGAG -3′; reverse, 5′-CTCTGTAGGTAGTTTGTCCAATTATGTC-3′; 341 bp). Non-transgenic littermates were used as controls for both transgenic lines. Mice were fed ad lib and kept under a 12-hour light/dark cycle.

### Metabolic analyses

Hyperinsulinemic-euglycemic clamps were performed in awake mice fasted for 5 hour as previously described [45] with modifications. Experiments used primed-continuous infusion of [3-^3^H] glucose (2.5 µCi bolus, 0.05 µCi/minute during the basal state, and 0.1 µCi/minute during the clamp period). Insulin was infused at the rate of 2.5 mU/kg/minute (without bolus). Indirect calorimetry and activity measurements were performed as described [46]. Lipid oxidation was carried out on conscious animals [47] and on isolated soleus muscle [45] as described. Body composition of 5–7 month old animals was measured using the EchoMRI 3-in-1™ (Echo Medical Systems).

### High-fat diet treatment and analysis

Nine week old males were fed either standard chow (NIH-31 Open Formula, Zeigler) or a 4.73 kcal/g HFD with 45% kcal from fat and 35% kcal from carbohydrates (D12451, Research Diets) for 10 weeks. GTT and ITT were performed after 8 and 9 weeks, respectively (see below). After 10 weeks, fed animals were anesthetized by i.p. injection of 80 mg/kg BW pentobarbital, and serum samples were collected by cardiac puncture. Liver and adipose samples were quickly frozen on dry ice or put in RNA Later (Ambion) for protein and RNA analysis.

### Glucose tolerance and insulin tolerance tests

GTT and ITT were performed as described [20]. Blood glucose was measured by tail bleeding at indicated time points using an Elite Glucometer (Bayer). Blood from tail tips of fasted mice was taken before and 30 minutes after injection of 2 mg/kg BW glucose for measurements of serum insulin during glucose challenge.

### RNA isolation, Northern blotting, and quantitative RT-PCR

Total RNA was isolated from tissues using Trizol Reagent (Invitrogen) according to the manufacturer's instructions. For Northern analysis, 20 µg total RNA was used for electrophoresis and blotting to GeneScreen Plus membrane (Perkin Elmer) and hybridized according to the manufacturer's instructions with a probe corresponding to the extracellular region of mouse *Acvr2b*. For real-time quantitative PCR, samples were digested with DNase followed by reverse transcription of 500 ng total RNA using Superscript II (Invitrogen). The resulting cDNA was quantified by real-time quantitative PCR on an ABI Prism® 7000 Sequence Detection System using indicated Taqman primers (Applied Biosystems) and normalized to *18S* expression.

### Serum measurements

Serum insulin, leptin, resistin, and glucagon were determined by radioimmunoassay (Millipore). Serum triglycerides (Infinity triglycerides kit, Thermo), free fatty acids (half micro test kit, Roche), β-hydroxybutyrate (Stanbio Laboratory), and lactate (BioVision) were determined by colorimetric methods according to the manufacturer's instructions.

### Phospho-Akt detection

For in vivo Akt detection, mice were fasted overnight, anesthetized with pentobarbital (80 mg/kg body weight), and injected i.p. with 10 U/kg BW insulin (Lilly). Gastrocnemius muscle, gonadal WAT and intrascapular BAT were removed after 10 minutes and frozen in a dry ice ethanol bath, homogenized in RIPA buffer (50 mM Tris-HCl pH 7.4, 1% NP-40, 0.25% sodium deoxycholate, 150 mM sodium chloride, 1 mM EDTA) containing 1 mM sodium orthovanadate, 1 mM sodium fluoride, and proteinase inhibitor cocktail (Roche), and centrifuged at 13,000 g for 10 minutes to collect supernatants. Supernatants (20 µg protein) were used for reducing SDS PAGE and Western blotting. For immunoblotting analysis of phospho-Akt, Western blots were incubated with a 1∶2000 dilution of rabbit anti-phospho-Akt-Ser473 (Cell Signaling), followed by a 1∶5000 dilution of HRP-conjugated goat anti-rabbit secondary antibody (Invitrogen), and detected by ECL (Pierce). Blots were then stripped (H_2_O, 0.2N sodium hydroxide, H_2_O, for 5 minutes each) and incubated with a 1∶2000 dilution of rabbit anti-Akt primary antibody (Cell Signaling) followed by detection as above to detect total Akt.

### Liver triglyceride measurements

Extraction of tissue triglycerides with chloroform/methanol was modified from Burant et al [48]. After hydrolysis with 200 µL of 3 M potassium hydroxide in 65% ethanol, triglycerides were measured radiometrically using a glycerol kinase assay [49].

### Histology

Tissues were dissected, fixed in 10% formalin, dehydrated, embedded in paraffin, sectioned, and stained with H&E.

### Statistical analysis

Results are expressed as mean±SEM. Statistical analysis of the hyperinsulinemic-euglycemic clamp data for increases in glucose infusion rate and glucose uptake in *Mstn ^−/−^* mice was performed using one-tailed student's *t* test. Statistical analysis of data from HFD experiments, except GTT and ITT, was performed using SPSS software by two-factor ANOVA with genotype and diet as the factors. When between-subjects effects were significant, data were further analyzed by pairwise comparisons using Bonferroni confidence interval adjustment for multiple comparisons. Indirect calorimetry, GTT, and ITT data were analyzed by 2-tailed student's *t* test. *P*<0.05 was considered statistically significant.

## Supporting Information

Figure S1 Fat pad mass of Mstn+/+ and Mstn−/− mice on standard chow or HFD.
(0.10 MB PDF)

Figure S2 Representative images of H&E stained gonadal fat pads from mutant mice and control littermates on standard chow or high fat diet (HFD).
(0.99 MB PDF)

Figure S3 Northern blot analysis of expression of muscle-DN transgene and Gapdh loading control from non-transgenic (−) and transgenic (+) mice.
(0.12 MB PDF)

Table S1 Indirect calorimetry
(0.05 MB PDF)

Table S2 Liver triglyceride concentration on standard or HFD
(0.11 MB PDF)
