# Supplementary material for: Myostatin Inhibition in Muscle, but Not Adipose Tissue, Decreases Fat Mass and Improves Insulin Sensitivity
Source: PLoS One. 2009 Mar 19;4(3):e4937. doi: 10.1371/journal.pone.0004937 (PMC2654157; doi:10.1371/journal.pone.0004937)
Supplement: Figure S1 — Fat pad mass of Mstn+/+ and Mstn−/− mice on standard chow or HFD. (0.10 MB PDF) [file pone.0004937.s001.pdf]

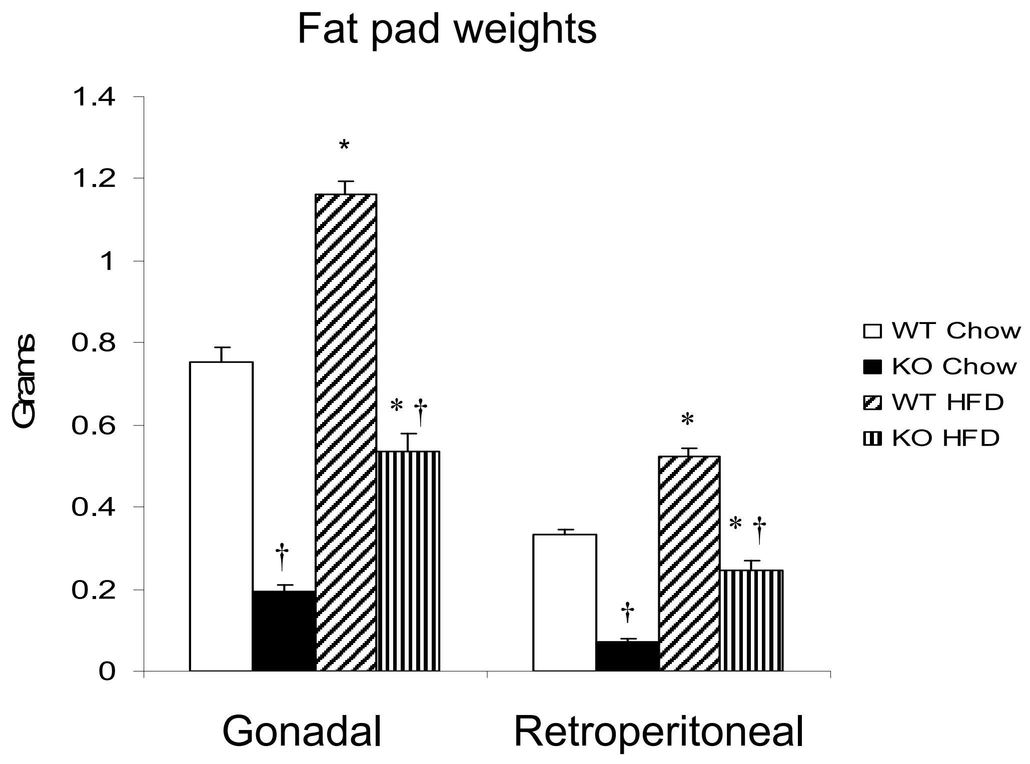

Figure S1

Fat pad mass of *Mstn*$^{+/+}$ and *Mstn*$^{-/-}$ mice on standard chow or HFD. $n = 7\text{-}16$. $^{*}P < 0.001$ vs. same genotype on standard chow. $^{\dagger}P < 0.001$ vs. WT control on same diet.
