# Supplementary material for: Myostatin Inhibition in Muscle, but Not Adipose Tissue, Decreases Fat Mass and Improves Insulin Sensitivity
Source: PLoS One. 2009 Mar 19;4(3):e4937. doi: 10.1371/journal.pone.0004937 (PMC2654157; doi:10.1371/journal.pone.0004937)
Supplement: Figure S2 — Representative images of H&E stained gonadal fat pads from mutant mice and control littermates on standard chow or high fat diet (HFD). (0.99 MB PDF) [file pone.0004937.s002.pdf]

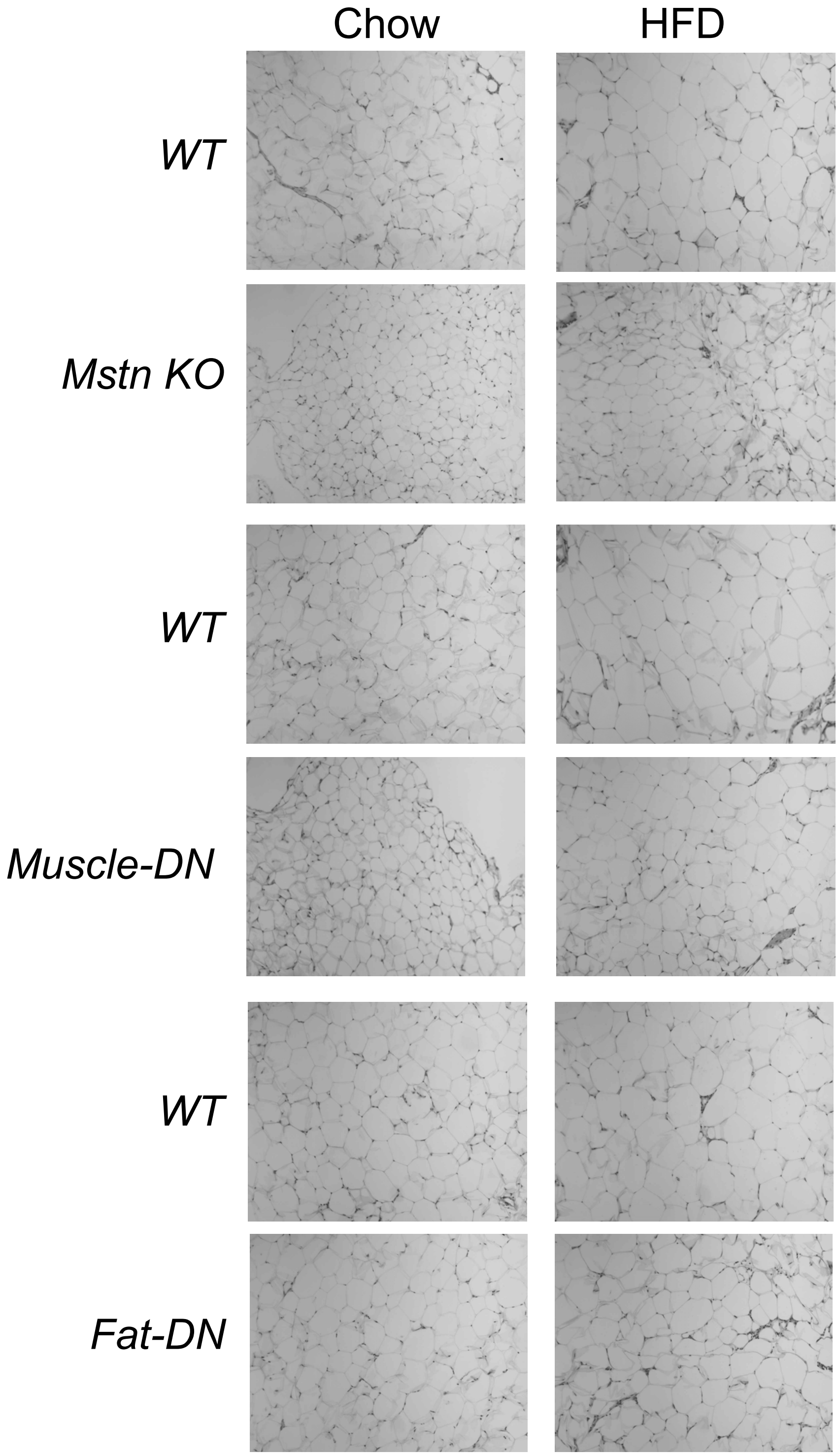

Figure S2

Representative images of H&E stained gonadal fat pads from mutant mice and control littermates on standard chow or high fat diet (HFD). *Mstn*$^{-/-}$ and *muscle-DN* mice, but not *fat-DN* mice, have smaller adipocytes compared to control mice on both diets.
