# Supplementary material for: Myostatin Inhibition in Muscle, but Not Adipose Tissue, Decreases Fat Mass and Improves Insulin Sensitivity
Source: PLoS One. 2009 Mar 19;4(3):e4937. doi: 10.1371/journal.pone.0004937 (PMC2654157; doi:10.1371/journal.pone.0004937)
Supplement: Figure S3 — Northern blot analysis of expression of muscle-DN transgene and Gapdh loading control from non-transgenic (−) and transgenic (+) mice. (0.12 MB PDF) [file pone.0004937.s003.pdf]

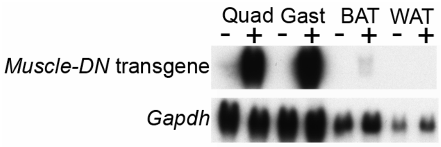

Figure S3

Northern blot analysis of expression of *muscle-DN* transgene and *Gapdh* loading control from non-transgenic (-) and transgenic (+) mice. Faint transgene expression was seen in brown adipose tissue (BAT). Quad, quadriceps muscle; Gast, gastrocnemius muscle; WAT, white adipose tissue. Twenty micrograms total RNA was run per lane.
