# Supplementary material for: Myostatin Inhibition in Muscle, but Not Adipose Tissue, Decreases Fat Mass and Improves Insulin Sensitivity
Source: PLoS One. 2009 Mar 19;4(3):e4937. doi: 10.1371/journal.pone.0004937 (PMC2654157; doi:10.1371/journal.pone.0004937)
Supplement: Table S1 — Indirect calorimetry (0.05 MB PDF) [file pone.0004937.s004.pdf]

Table S1
Indirect calorimetry

	Mstn$^{+/+}$	*Mstn*$^{-/-}$	***P* value**
Body weight (g)	32.7 ± 0.8	35.3 ± 0.4	0.028
Lean mass (g)	23.1 ± 0.4	31.7 ± 0.3	<0.001
Fat mass (g)	9.5 ± 0.6	3.3 ± 0.4	<0.001

	**23°C**			**30°C**		
	Mstn$^{+/+}$	*Mstn*$^{-/-}$	***P* value**	*Mstn*$^{+/+}$	*Mstn*$^{-/-}$	***P* value**
Energy expenditure (kcal/hr)						
Resting	0.56 ± 0.01	0.63 ± 0.01	<0.001	0.37 ± 0.01	0.43 ± 0.01	0.002
Total	0.66 ± 0.01	0.73 ± 0.01	0.001	0.48 ± 0.02	0.54 ± 0.01	0.031
Resting vO_2 (mL/hr)	114.8 ± 2.5	128.6 ± 1.3	<0.001	74.5 ± 2.3	86.8 ± 2.0	0.002
Total vO_2 (mL/hr)	141.3 ± 3.9	146.3 ± 3.9	0.390	102.7 ± 4.5	105.9 ± 2.8	0.599
Resting vO_2 (mL/hr/lean mass$^{0.75}$)	10.9 ± 0.2	9.6 ± 0.1	<0.001	7.1 ± 0.2	6.5 ± 0.2	0.055
Total vO_2 (mL/hr/lean mass$^{0.75}$)	13.4 ± 0.3	11.0 ± 0.3	<0.001	9.7 ± 0.4	7.9 ± 0.2	0.003
Resting RER (vCO_2/vO_2)	0.837 ± 0.016	0.861 ± 0.017	0.354	0.871 ± 0.019	0.935 ± 0.013	0.024
Total RER (vCO_2/vO_2)	0.843 ± 0.015	0.889 ± 0.008	0.035	0.862 ± 0.017	0.945 ± 0.012	0.003
Activity (beam breaks/min)						
Total	185.2 ± 29.8	143.2 ± 30.7	0.355	159.4 ± 21.7	119.6 ± 12.0	0.176
Ambulating	57.2 ± 14.7	45.6 ± 12.2	0.578	44.2 ± 9.5	27.9 ± 4.8	0.200

All data are expressed as mean ± SEM (*Mstn*$^{+/+}$, n = 10 and *Mstn*$^{-/-}$, n = 7). P values < 0.05 were considered significant.

Table S2
Liver triglyceride concentration on standard or HFD

	Liver triglyceride (µmol/g)	*P* value (between diets within a genotype)	*P* value (versus control within a diet)
**Standard Chow**			
Mstn$^{+/+}$	22.5 ± 2.4		
Mstn$^{-/-}$	6.1 ± 1.0		0.036
**HFD**			
Mstn$^{+/+}$	73.3 ± 6.3	<0.001	
Mstn$^{-/-}$	24.8 ± 3.0	0.004	<0.001
**Standard Chow**			
Non-transgenic	18.6 ± 1.2		
Muscle-DN	11.4 ± 1.1		1.000
**HFD**			
Non-transgenic	23.3 ± 2.3	0.282	
Muscle-DN	16.2 ± 1.1	0.341	1.000
**Standard Chow**			
Non-transgenic	19.9 ± 1.5		
Fat-DN	20.4 ± 2.0		1.000
**HFD**			
Non-transgenic	50.8 ± 5.1	<0.001	
Fat-DN	40.8 ± 3.6	<0.001	0.321

Data are expressed as mean ± SEM of 4-11 per group.
