# Supplementary material for: Myostatin Inhibition in Muscle, but Not Adipose Tissue, Decreases Fat Mass and Improves Insulin Sensitivity
Source: PLoS One. 2009 Mar 19;4(3):e4937. doi: 10.1371/journal.pone.0004937 (PMC2654157; doi:10.1371/journal.pone.0004937)
Supplement: Table S2 — Liver triglyceride concentration on standard or HFD (0.11 MB PDF) [file pone.0004937.s005.pdf]

**Table S2**
Liver triglyceride concentration on standard or HFD

| | **Liver triglyceride (µmol/g)** | ***P* value** (between diets within a genotype) | ***P* value** (versus control within a diet) |
|---|---|---|---|
| **Standard Chow** | | | |
| *Mstn*$^{+/+}$ | 22.5 ± 2.4 | | |
| *Mstn*$^{-/-}$ | 6.1 ± 1.0 | | 0.036 |
| **HFD** | | | |
| *Mstn*$^{+/+}$ | 73.3 ± 6.3 | <0.001 | |
| *Mstn*$^{-/-}$ | 24.8 ± 3.0 | 0.004 | <0.001 |
| | | | |
| **Standard Chow** | | | |
| *Non-transgenic* | 18.6 ± 1.2 | | |
| *Muscle-DN* | 11.4 ± 1.1 | | 1.000 |
| **HFD** | | | |
| *Non-transgenic* | 23.3 ± 2.3 | 0.282 | |
| *Muscle-DN* | 16.2 ± 1.1 | 0.341 | 1.000 |
| | | | |
| **Standard Chow** | | | |
| *Non-transgenic* | 19.9 ± 1.5 | | |
| *Fat-DN* | 20.4 ± 2.0 | | 1.000 |
| **HFD** | | | |
| *Non-transgenic* | 50.8 ± 5.1 | <0.001 | |
| *Fat-DN* | 40.8 ± 3.6 | <0.001 | 0.321 |

Data are expressed as mean ± SEM of 4-11 per group.
